# A new species of *Apolochus* (Crustacea, Amphipoda, Gammaridea, Amphilochidae) in Maryland coastal bays, USA with notes on its abundance and distribution

**DOI:** 10.3897/zookeys.571.7440

**Published:** 2016-03-07

**Authors:** Andrés G. Morales-Núñez, Paulinus Chigbu

**Affiliations:** 1 NSF – CREST Center for the Integrated Study of Coastal Ecosystem Processes and Dynamics in the Mid-Atlantic Region (CISCEP); 2 NOAA Living Marine Resources Cooperative Science Center (LMRCSC), Department of Natural Sciences, University of Maryland Eastern Shore, Princess Anne, MD 21853, USA

**Keywords:** Amphipoda, Amphilochidae, *Apolochus*, new species, Maryland coastal bays (MCBs), Mid-Atlantic Region, Anfipoda, Amphilochidae, *Apolochus*, nueva especie, bahías costeras de Maryland, región del Atlántico medio

## Abstract

A new amphilochid amphipod, *Apolochus cresti* **sp. n.** is described from specimens collected in the shallow waters of Maryland coastal bays, Mid-Atlantic region, at depths from 1.7 to 2.1 m. The new species appears to be most closely related to the northeastern Atlantic species, *Apolochus neapolitanus* *sensu* Krapp-Schickel, 1982. *Apolochus cresti* **sp. n.** can be distinguished from *Apolochus neapolitanus* by a combination of characters, including the shape of the lateral cephalic lobe, shape of the mandible molar process, relative length of mandible palp article 3, the carpal lobe length of gnathopod 2, and the lack of sub-marginal spines on antero-lateral surface of gnathopod 2. Spearman’s rank correlation analysis indicated a positive correlation between the abundance of *Apolochus cresti* and the amount of macroalgae collected per station, bay, and month. Ovigerous females carrying eggs were present from March to May and in October, reaching their peak in May, although only ovigerous females carrying juveniles were found in May. Males were abundant in March and were collected also in May and October. A key for the separation of *Apolochus* species is presented.

## Abstract (es)

Una nueva especie de anfípodo, *Apolochus cresti* **sp. n.** es descrita con especímenes colectados en aguas someras de las bahías de Maryland, en la región del Atlántico medio a una profundidad entre 1.7 a 2.1 m. La nueva especie parece estar más cercanamente relacionada a la especie *Apolochus neapolitanus* *sensu* Krapp-Schickel, 1982 del Atlántico este. *Apolochus cresti* **sp. n.** puede ser distinguida de *Apolochus neapolitanus* por una combinación de caracteres, incluyendo la forma del lóbulo lateral cefálico, la forma del proceso molar de la mandíbula, la longitud relativa proporción del artículo 3 del palpo mandibular, la longitud del lóbulo del carpo del gnatópodo 2, y la falta de espinas sub-marginales en la parte antero-lateral del gnatópodo 2. El análisis del coeficiente de correlación de Spearman indica una correlación positiva entre la abundancia de *Apolochus cresti* y la cantidad de macro-algas colectadas por estación, y bahía, y mes. Hembras ovígeras cargando huevos fueron encontradas desde marzo a mayo y en octubre, alcanzando su pico en mayo; aunque solo hembras ovígeras cargando juveniles fueron encontradas en mayo. Machos fueron abundantes en marzo y fueron colectados también en mayo y octubre. Una clave para la separación de especies de *Apolochus* es presentada.

## Introduction

During a survey of benthic marine macro-invertebrates of the Maryland coastal bays (MCBs), hundreds of specimens of an undescribed species, belonging to the family Amphilochidae, with attributes of the genus *Apolochus* Hoover & Bousfield, 2001, were discovered among the amphipods collected. Amphilochids are very common in the marine benthic fauna, associated with macroalgae or as inquilines and commensals with sea fans, hydroids and other sessile marine invertebrates (Hoover and Bousfield 2001, Leite 2002, Azman 2009, Leite and Siqueira 2013). Members of this family are often overlooked because of their small size (McKinney 1978) and most of the specimens are extremely fragile and brittle (Azman 2009). In this study, a new species of *Apolochus* from MCBs is described and information on its abundance and distribution is provided. To date, the genus *Apolochus* contains eight species (WoRMS). Four species have been reported from the Atlantic Ocean: *Apolochus casahoya* (McKinney, 1978) and *Apolochus delacaya* (McKinney, 1978) from the Gulf of Mexico (McKinney 1978), Florida Keys (Barnard and Thomas 1983) and Venezuela (Martin et al. 2013); *Apolochus neapolitanus* (Della Valle, 1893) with a pantropical distribution from the Mediterranean (Della Valle 1893), Gulf of Mexico (McKinney 1978), Florida Keys (Thomas 1993), Venezuela (Martin et al. 2013) and Brazil (Wakabara and Serejo 1998); and *Apolochus pillaii* (Barnard & Thomas, 1983) from the Florida Keys (Barnard and Thomas 1983) and Gulf of Mexico (Paz-Ríos and Ardisson 2013). Four additional species have been reported from the eastern North Pacific: *Apolochus barnardi* Hoover & Bousfield, 2001 from central to southern California (Hoover and Bousfield 2001); *Apolochus litoralis* (Stout, 1912) from southern California, Oregon, Washington, British Columbia, and southern Alaska (Stout 1912, Hoover and Bousfield 2001); *Apolochus picadurus* (Barnard, 1962) from southern California (Barnard 1962); and *Apolochus staudei* Hoover & Bousfield, 2001 from British Columbia (Hoover and Bousfield 2001). Additionally, LeCroy (2002) reported the presence of an undescribed species of *Apolochus*, *Apolochus* sp. A., from the Gulf of Mexico and Florida Keys, a species probably misidentified in this region as *Apolochus neapolitanus*. It is worthwhile to note that all illustrations indicating the presence of *Apolochus neapolitanus* in different parts of the world show morphological differences, indicating that they are not the same species (LeCroy 2002). Unfortunately, the original description of *Apolochus neapolitanus* is poor and incomplete (Della Valle 1883).

## Materials and methods

**Figure 1.**
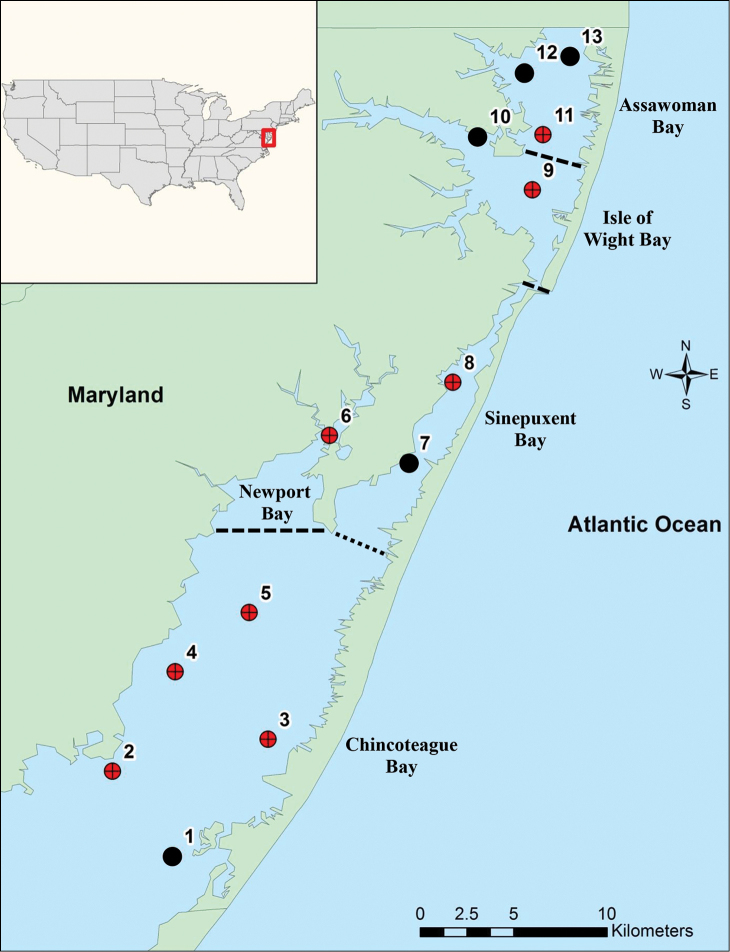
Map of Maryland Coastal Bays indicating the 13 stations sampled. Black circles indicate the five stations where *Apolochus cresti* sp. n., was found.

The material examined was collected in the MCBs, mid-Atlantic region, on the east coast of the United States of America. The MCBs consist of five principal lagoons distributed in two areas. Assawoman and Isle of Wight Bays are located in the northern area of the MCBs; and Sinepuxent, Newport, and Chincoteague Bays are located in the southern area of the MCBs (Figure 1). These five bays are different with respect to depth, water flow, area, and level of anthropogenic impact. In general, the MCBs are shallow systems with an average depth of 1.2 m and are predominantly polyhaline, with salinities greater than 25 PSU. Additionally, the surface areas vary from 15.9 km^2^ in Newport Bay to 189 km^2^ in Chincoteague Bay (Chaillou et al. 1996; Wazniak et al. 2004).

Samples were taken at 13 stations (eight stations in the southern area and five in the northern area) (Fig. 1). Sampling was conducted monthly for nine months from March to December 2012, although due to inclement weather conditions, samples were not collected in September. Samples were collected using an epibenthic sled (area = 0.39 m²), with a 1 mm mesh size, to which was attached a flow meter Model 2030R (General Oceanics). Field sampling was completed in two days each month. At each station, two horizontal tows were conducted at an average speed of 2 knots for 5 min. In the field, the net was rinsed and all macroinvertebrates were passed through a 0.5 mm sieve. After sieving, all macroinvertebrates were fixed in 5% neutral buffered formalin. Additionally, the epifauna was separated from the macroalgae by shaking each macroalgal fragment in a bucket filled with seawater. The macroinvertebrates retained were passed through a 0.5 mm sieve and fixed in 5% neutral buffered formalin. All macroalgae collected with the sled were stored in plastic bags with seawater in a cooler. Subsequently, the wet weight of the macroalgae collected at each site was determined in the laboratory, and then the macroalgae and any remaining associated epifauna were preserved in 5% neutral buffered formalin. Finally, the macroalgae were washed over a sieve with a 0.3 mm mesh size. Each macroalgal fragment was then visually examined further to confirm that all epifaunal invertebrates were removed. All amphipods collected were counted, identified to the lowest practical taxonomic level and preserved in ethanol (70%). Thereafter, the macroalgae were identified to the lowest practical taxonomic level.

Water quality data were collected in situ using a YSI 6600 Multi-Parameter Water Quality Sonde and included water temperature, salinity, dissolved oxygen, and pH, which were all recorded at 0.3 m from the bottom (Tables 1–2). Additionally, water depth and clarity (i.e. Secchi disc transparency) were recorded at each station.

**Table 1.** Mean values of abiotic variables ± SE per station from March to December 2012 in the Maryland Coastal Bays.

Stations	Temperature (°C)	Salinity (PSU)	Dissolved Oxygen (mg/L)	pH	Depth (m)	Secchi depth (m)
1	17.8 ± 2.24	33.3 ± 1.09	8.1 ± 0.45	8.0 ± 0.03	2.1 ± 0.08	0.6 ± 0.14
2	17.9 ± 3.35	30.9 ± 1.67	8.0 ± 0.74	7.9 ± 0.04	1.1 ± 0.23	0.6 ± 0.22
3	16.8 ± 2.16	32.2 ± 1.23	8.3 ± 0.47	8.0 ± 0.03	2.0 ± 0.13	0.7 ± 0.17
4	16.5 ± 2.16	31.2 ± 0.99	8.0 ± 0.57	7.9 ± 0.04	2.0 ± 0.29	0.6 ± 0.14
5	16.5 ± 2.18	31.5 ± 1.19	8.3 ± 0.49	8.0 ± 0.02	1.8 ± 0.09	0.8 ± 0.20
6	17.5 ± 2.11	27.9 ± 1.52	8.1 ± 0.65	7.9 ± 0.07	1.1 ± 0.17	0.7 ± 0.55
7	17.5 ± 2.65	32.9 ± 1.25	7.4 ± 0.71	7.9 ± 0.05	1.9 ± 0.33	0.9 ± 0.20
8	16.5 ± 2.31	34.0 ± 0.88	7.7 ± 0.61	7.9 ± 0.04	2.4 ± 0.35	0.8 ± 0.22
9	16.0 ± 2.18	33.4 ± 0.94	7.9 ± 0.62	7.9 ± 0.05	2.0 ± 0.29	0.8 ± 0.17
10	17.5 ± 2.23	30.7 ± 0.99	8.0 ± 0.68	8.0 ± 0.05	1.8 ± 0.05	0.7 ± 0.20
11	18.0 ± 3.12	30.1 ± 1.37	7.7 ± 0.98	7.9 ± 0.11	3.1 ± 0.05	1.1 ± 0.27
12	17.2 ± 2.24	29.8 ± 1.17	8.1 ± 0.74	8.0 ± 0.07	1.7 ± 0.07	0.8 ± 0.18
13	17.3 ± 2.28	29.5 ± 1.35	7.8 ± 0.67	7.9 ± 0.08	2.1 ± 0.05	1.1 ± 0.23

**Table 2.** Mean values of abiotic variables ± SE per bay from March to December 2012 in the Maryland Coastal Bays.

Areas	Temperature (°C)	Salinity (PSU)	Dissolved Oxygen (mg/L)	pH	Depth (m)	Secchi depth (m)
Assawoman Bay	17.5 ± 1.37	29.8 ± 0.72	7.9 ± 0.43	7.9 ± 0.05	2.2 ± 0.12	1.1 ± 0.31
Isle of Wight Bay	16.7 ± 1.52	32.1 ± 0.74	7.9 ± 0.44	7.9 ± 0.04	1.9 ± 0.14	0.9 ± 0.30
Sinepuxent Bay	16.9 ± 1.69	33.5 ± 0.72	7.6 ± 0.45	7.9 ± 0.03	2.2 ± 0.24	1.0 ± 0.38
Newport Bay	17.5 ± 2.11	27.9 ± 1.52	8.1 ± 0.65	7.9 ± 0.07	1.1 ± 0.17	0.7 ± 0.55
Chincoteague Bay	17.0 ± 1.00	31.9 ± 0.53	8.1 ± 0.23	8.0 ± 0.07	1.8 ± 0.10	0.8 ± 0.19

The density of *Apolochus* sp. n. was estimated and expressed as number of individuals per m² (ind m^-2^), and the macroalgae biomass was expressed as gram wet weight per m^2^ (g ww m^-2^). Spearman’s rank correlations were calculated to determine whether the abundance and distribution of *Apolochus* sp. n. were related to the amount of macroalgae collected at the stations, bays, and within months during this study. Furthermore, Spearman’s rank correlations were calculated per stations, bays, and months between biotic (i.e. abundance of amphipods and biomass of macroalgae) and abiotic variables such as water temperature, salinity, dissolved oxygen, pH, depth, and Secchi depth.

Specimens of *Apolochus* sp. n. were dissected under an Olympus ZS-16 stereomicroscope. Appendages were mounted on glass slides in glycerin and observed with an Olympus BX41 compound microscope, and drawings were made with a camera lucida. Illustrations were prepared with Adobe Illustrator and Photoshop CS6 Extended. The classification of crustacean spines and setae follows Watling (1989) and Zimmer et al. (2009).

Type material has been deposited in the National Museum of Natural History, Smithsonian Institution, Washington DC (USNM) and the Gulf Coast Research Laboratory (GCRL) Museum, Ocean Springs, Mississippi MS. All measurements are in millimeteres (mm). Total body length (TL) was measured from the tip of the rostrum to the tip of the telson.

## Systematics

### Order Amphipoda Latreille, 1816 Suborder Gammaridea Latreille, 1802 Family Amphilochidae Boeck, 1871

#### Genus Apolochus

Taxon classificationAnimaliaAmphipodaAmphilochidae

Hoover & Bousfield, 2001

##### Type species.

*Amphilochus neapolitanus* Della Valle, 1893

##### Generic diagnosis.

See Hoover and Bousfield (2001).

#### Apolochus cresti sp. n.

Taxon classificationAnimaliaAmphipodaAmphilochidae

http://zoobank.org/56C24D6B-2E21-4711-928C-F63A291D0A33

Figs 2 , 3 , 4 , 5 , 6 , 7 , 8 ; 9H, V, Z, B–9 , 1−C-1

##### Material examined.

*Holotype*: ovigerous ♀ (USNM 1254651), 3.4 mm , station 10 (38°14.504'N; 75°09.306'W), Isle of Wight Bay, USA, 1.8 m, 17.5 °C, 30.7 PSU, 16 May 2012, coastal lagoon, coll. A.G. Morales-Núñez. *Paratypes*: 4 ♀♀ (USNM 1254652), 4 ♂♂ (USNM 1254653); 4 ♀♀ and 4 ♂♂ (GCRL 06537), same collection data as for holotype. Additional specimens from the type locality are in the collection of the authors.

##### Additional material.

587 specimens (558 undetermined, 11 non-ovigerous ♀♀, 1 ovigerous ♀, and 17 ♂♂), station 10 (38°14.504'N; 75°09.306'W), Isle of Wight Bay, 1.8 m, 17.5 °C, 30.7 PSU, 15 March 2012, coll. A.G. Morales-Núñez. − 327 specimens (313 undetermined and 14 ♂♂), station 12 (38°25.778'N; 75°05.956'W), Assawoman Bay, 1.7 m, 17.2 °C, 29.8 PSU, 15 March 2012, coll. A.G. Morales-Núñez. − 2 specimens (undetermined), station 1 (38°03.143'N; 75°16.114'W), Chincoteague Bay, 2.1 m, 17.8 °C, 33.3 PSU, 17 April 2012, coll. A.G. Morales-Núñez. − 1 specimen (ovigerous ♀), station 10 (38°14.504'N; 75°09.306'W), Isle of Wight Bay, 1.8 m, 17.5 °C, 30.7 PSU, 17 April 2012, coll. A.G. Morales-Núñez. − 15 specimens (6 undetermined and 9 ovigerous ♀♀), station 12 (38°25.778'N; 75°05.956'W), Assawoman Bay, 1.7 m, 17.2 °C, 29.8 PSU, 15 March 2012, coll. A.G. Morales-Núñez. − 8 specimens (2 undetermined, 1 non-ovigerous ♀, and 5 ovigerous ♀), station 13 (38°26.240'N; 75°04.651'W), Assawoman Bay, 2.1 m, 17.3 °C, 29.5 PSU, 17 April 2012, coll. A.G. Morales-Núñez. − 3 specimens (undetermined), station 7 (38°14.504'N; 75°09.306'W), Sinepuxent Bay, 1.9 m, 17.5 °C, 32.9 PSU, 21 May 2012, coll. A.G. Morales-Núñez. − 632 specimens (502 undetermined, 22 non-ovigerous ♀♀, 83 ovigerous ♀, and 25 ♂♂), station 10 (38°14.504'N; 75°09.306'W), Isle of Wight Bay, 1.8 m, 17.5 °C, 30.7 PSU, 21 May 2012, coll. A.G. Morales-Núñez. − 1 specimen (undetermined), station 12 (38°25.778'N; 75°05.956'W), Assawoman Bay, 1.7 m, 21 May 2012, coll. A.G. Morales-Núñez. − 552 specimens (517 undetermined, 28 ovigerous ♀, and 7 ♂♂), station 10 (38°14.504'N; 75°09.306'W), Isle of Wight Bay, 1.8 m, 17.5 °C, 30.7 PSU, 25 October 2012, coll. A.G. Morales-Núñez. − 9 specimens (5 undetermined and 4 ovigerous ♀), station 13 (38°26.240'N; 75°04.651'W), Assawoman Bay, 2.1 m, 17.3 °C, 29.5 PSU, 25 October 2012, coll. A.G. Morales-Núñez.

##### Diagnosis.

**Female**: *Antenna* 1 and 2 sub-equal in length; accessory flagellum uniarticulate, small (hard to see without higher magnification). Mandibular molar with row of spinules/setae running up the margin of the molar column, with three marginal spines on triturating surface. Mandible palp article 3 longer than two proximal articles. *Gnathopod 2* carpus with elongated lobe reaching along posterior margin of propodus to palmar angle; propodus without sub-marginal spines on antero-lateral surface.

##### Etymology.

Named in honour of NSF – Centers of Research Excellence in Science and Technology (CREST), in recognition of its support to promote the development of new knowledge.

##### Type locality.

Isle of Wight Bay (38°14.504'N; 75°09.306'W), Maryland Coastal Bays, United States of America.

##### Distribution.

Maryland Coastal Bays, Mid-Atlantic region, USA, at depths ranging from 1.7 to 2.1 m.

##### Description.

**Figure 2.**
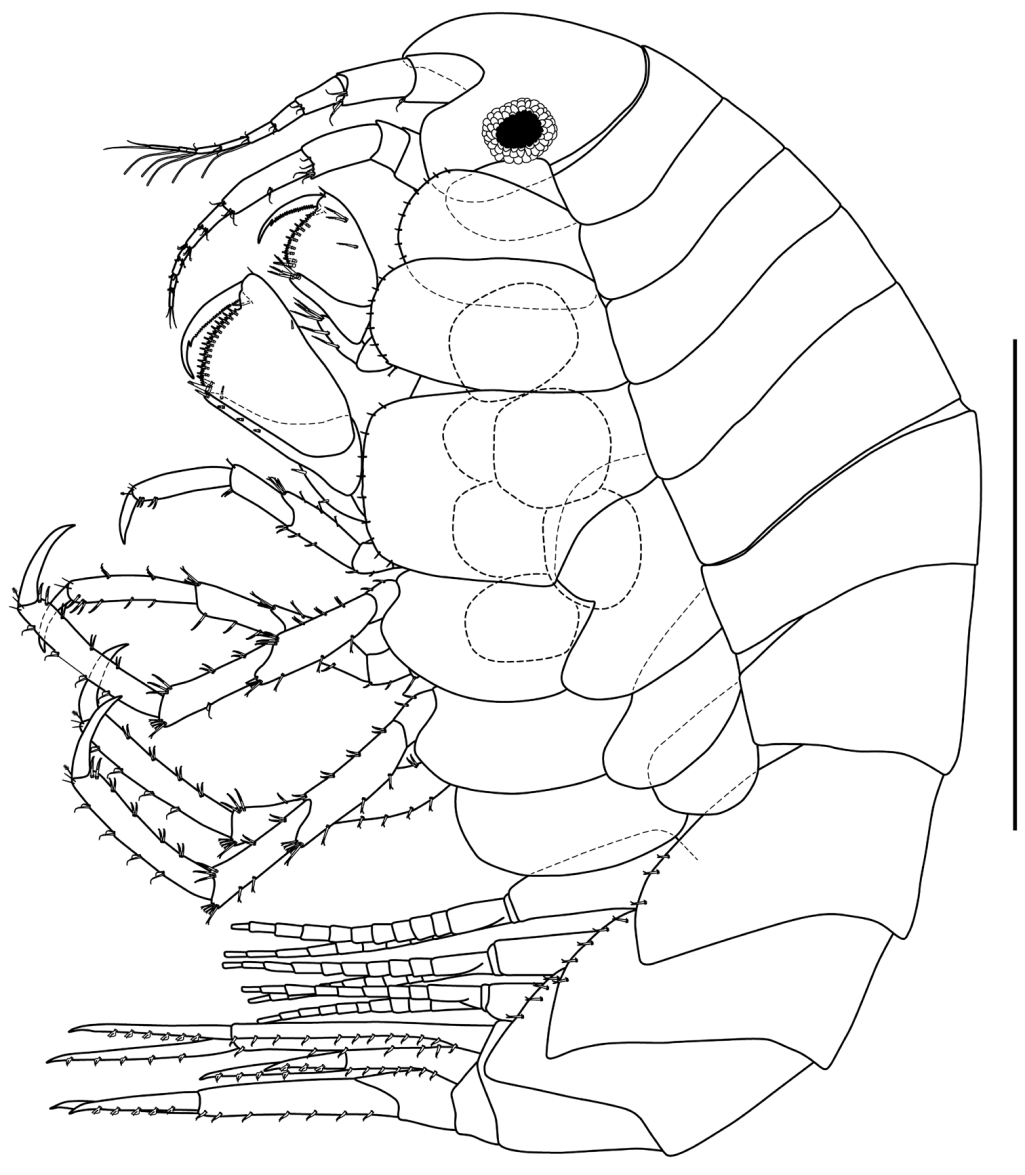
*Apolochus cresti* sp. n., Holotype female: lateral view. Scale bar: 1.0 mm.

**Ovigerous female (eight eggs).** *Body* (Fig. 2): Length 3.4 mm.

*Head* (Fig. 2): approximately 15% TL, slightly shorter than pereonites 1−3 combined; lateral cephalic lobe rounded; rostrum strong, downturned. Eyes circular with black center bordered by numerous opaque ommatidia.

**Figure 3.**
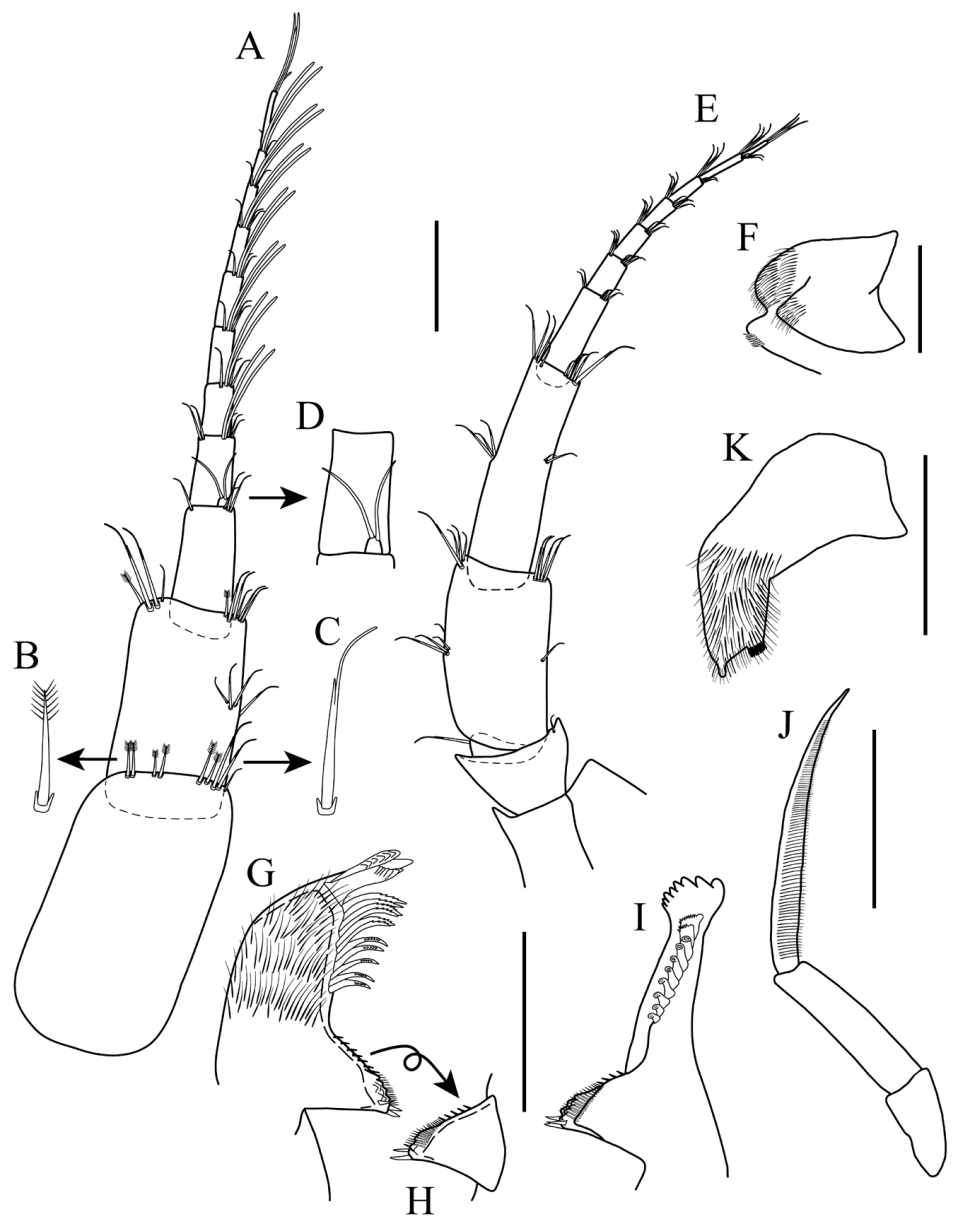
*Apolochus cresti* sp. n., Holotype female: **A** antenna 1, lateral view **B** enlargement of plumose seta **C** enlargement of cuspidate seta **D** enlargement of accessory flagellum **E** antenna 2, lateral view **F** upper lip **G** left mandible **H** opposite view of molar process of left mandible **I** right mandible **J** mandible palp **K** one side of lower lip. Scale bars: 0.1 mm (**A, E–J**).

*Antenna 1* (Fig. 3A−D): sub-equal to antenna 2, slightly shorter than head and pereonites 1−3 combined, peduncle shorter than head. Peduncle article 1 length approximately 1.3 times as long as wide, with four plumose setae (Fig. 3B) on dorso-distal margin, with five (two plumose and three cuspidate (Fig. 3C) setae on ventro-distal margin. Peduncle article 2 length approximately 1.4 times as long as wide, with four (one plumose and three cuspidate) setae on dorso-distal margin; with a cluster of four cuspidate setae on mid-ventral margin, and six (one plumose and five cuspidate) setae disto-ventrally. Peduncle article 3 length approximately 2.2 times as long as wide, with cuspidate seta on dorso-distal margin; with three cuspidate setae on ventro-distal margin. Flagellum with 6 to 8 articles, longer than peduncle, ventro-distal margin of each article with 3 to 4 cuspidate setae, first seven articles bearing two aesthetascs, article 8 with four simple setae of varying lengths apically. Accessory flagellum uniarticulate, small (difficult to see in low magnification), with two apical simple setae, length approximately ⅛ that of peduncle article 3 (Fig. 3D).

*Antenna 2* (Fig. 3E): sub-equal to antenna 1, peduncle slightly longer than head. Peduncle article 4 length approximately 1.7 times as long as wide, with two clusters (middle and distal) of cuspidate setae of varying lengths on dorsal and ventral margins. Peduncle article 5 length approximately 3.5 times as long as wide, with two clusters (middle and distal) of cuspidate setae of varying lengths on dorsal and ventral margins. Flagellum with seven articles, shorter than peduncle; first six articles with 2 to 5 cuspidate setae each, article 7 with three simple setae of sub-equal length apically.

*Mouthparts*: *Upper lip* (Fig. 3F): bilobed, densely pubescent apically.

*Mandibles* (Fig. 3G–J): molar broadly conical, with row of spinules/setae running up margin of molar column, with three marginal spines on triturating surface (Fig. 3G−I). Left mandible, densely setose, spine row with 11 to 13 accessory blades; blades increasing in width distally; *lacinia mobilis* with six teeth, incisor process dentate. Right mandible, densely setose, spine row with 10 accessory blades, incisor process dentate. *Palp*: with three articles; article 1 asetose, length less than half of article 2; article 2 asetose, length more than twice of length of article 1; article 3 longest, lanceolate, twice length of article 2, with dense row of longitudinal fine comb setae on medial surface (Fig. 3J).

*Lower lip* (Fig. 3K): bilobed, outer lobe densely setose, with apical gap, distal inner margin with serrate lobe, outer margin with tubercle.

**Figure 4.**
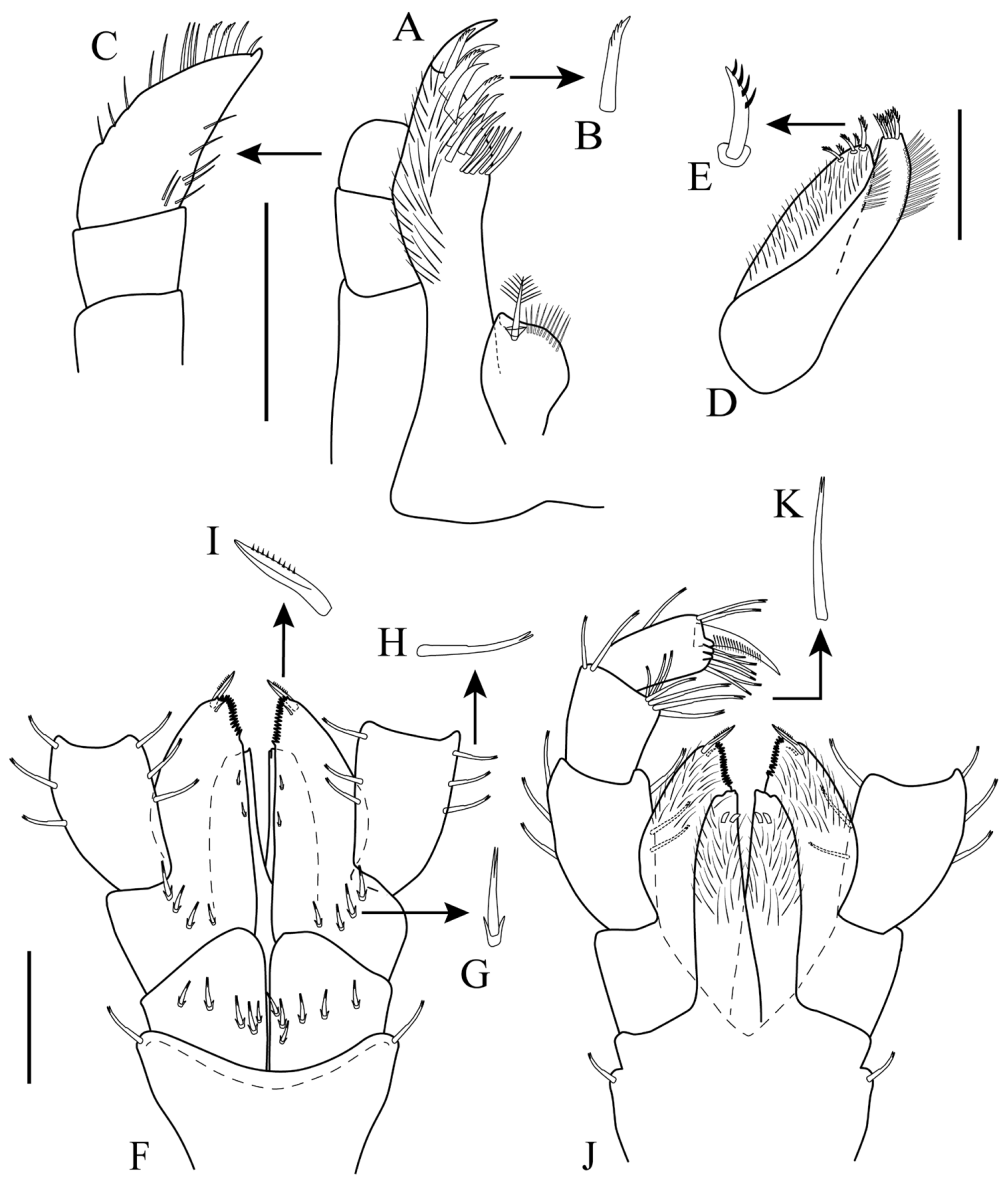
*Apolochus cresti* sp. n., Holotype female: **A** maxilla 1 **B** enlargement of serrate spine **C** maxilla 1 palp **D** maxilla 2 **E** enlargement of serrate spine **F** maxilliped, ventral view **G** enlargement of short bifurcate seta **H** enlargement of medium bifurcate seta **I** enlargement of well-developed serrate spine **J** maxilliped, dorsal view **K** enlargement of long bifurcate seta. Scale bars: 0.1 mm (**A, C−D, F, J**).

*Maxilla 1* (Fig. 4A−C): inner plate rounded, with apical plumose seta and row of simple setae on distal margin (Fig. 4A); outer plate with oblique distal margin bearing eleven robust spines (eight simple and three serrate (Fig. 4B), with five long slender setae on inner margin, outer margin densely setose (Fig. 4A). *Palp* (Fig. 4C): with two articles; article 1 asetose; article 2 length twice that of article 1, with two serrate (Fig. 4B) and two simple spines distally, mid-proximal inner margin and mid-distal outer margin with setae of different sizes.

*Maxilla 2* (Fig. 4D−E): inner plate densely setose, with five serrate (Fig. 4E) terminal setae of varying length, outer margin with row of simple setae; outer plate longer than inner, with four serrate terminal setae, outer margin with row of simple setae.

*Maxilliped* (Fig. 4F−K): inner plate densely setose, with two sub-distal tooth-like modified setae (Fig. 4J); outer plate setose distally, with four short bifurcate (Fig. 4G) setae proximally, a serrate inner distal margin, two small marginal spines, one well-developed serrate spine (Fig. 4I) and a small seta terminally (Fig. 4F−J). *Palp* (Fig. 4J−K): with four articles; article 1 longest; inner and outer margin with two to three medium bifurcate setae (Fig. 4H); article 2, approximately two-thirds length of article 1, with seven bifurcate setae on inner distal margin, two bifurcate setae on outer distal margin; article 3 slightly longer than article 2, with six (three medium bifurcate setae and three long bifurcate (Fig. 4K)) setae on inner distal margin, with three denticles distally, with three (one central and two distal) bifurcate setae on outer margin; dactylus shorter than article 3, without unguis, lanceolate, with dense longitudinal row of fine comb setae.

*Pereon* (Fig. 2): approximately 42% TL, pereonites 1−7 deeper than wide; pereonite 7 longest and widest.

**Figure 5.**
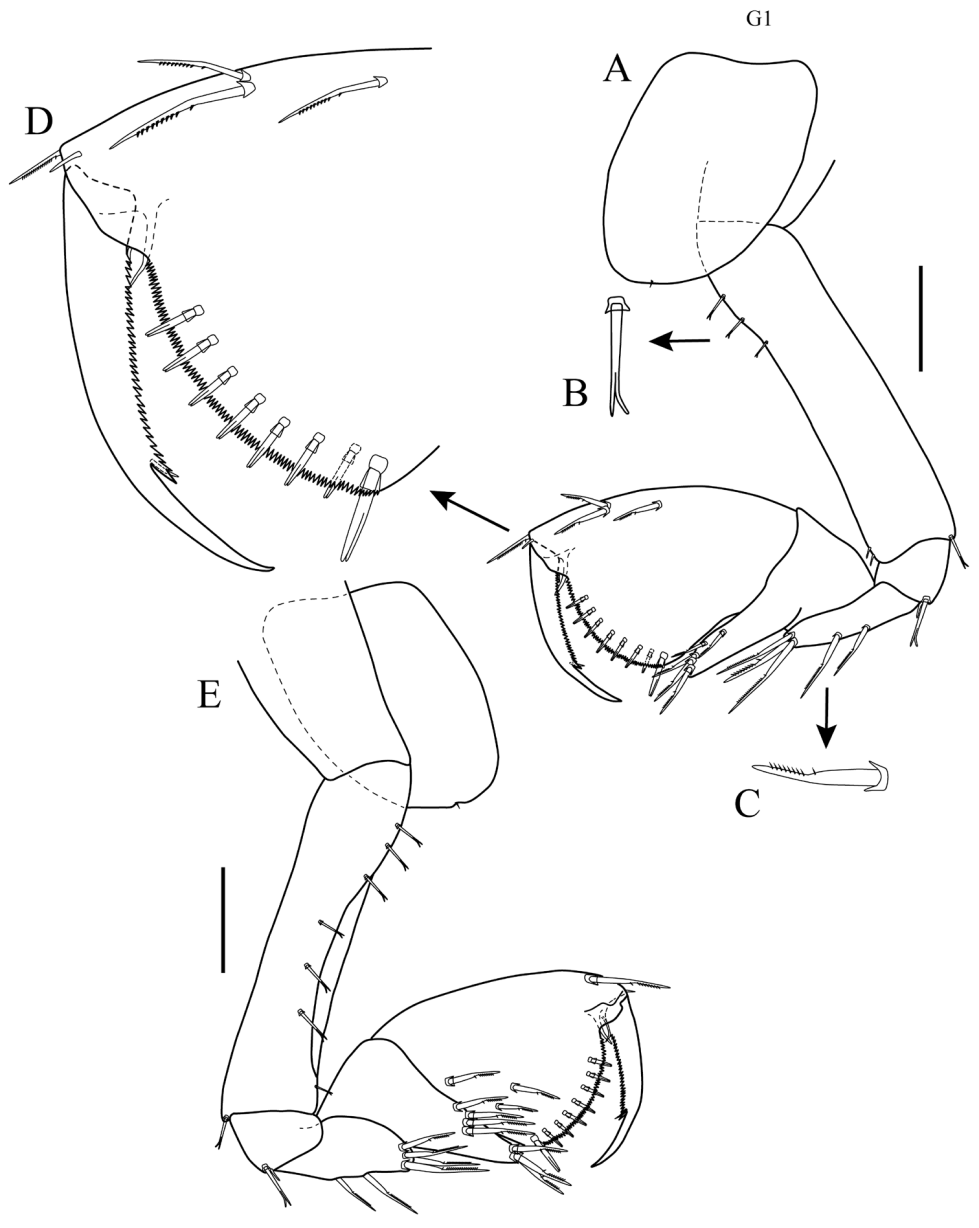
*Apolochus cresti* sp. n., Holotype female: **A** gnathopod 1, lateral view **B** enlargement of bifurcate seta **C** enlargement of serrate seta **D** enlargement of tip of propodus and dactylus of gnathopod 1 **E** gnathopod 1, medial view. Scale bar: 0.1 mm (**A, E**).

*Gnathopod 1*: *Lateral view* (Figs 3, 5A−D): subchelate; coxal plate short, sub-oval, partially hidden by coxa 2 (Fig. 3), with one marginal seta. Basis approximately 4.5 times as long as wide, anterior margin with three proximal bifurcate setae, two distal simple setae (Fig. 5B); postero-distal margin with one bifurcate seta. Ischium wider than long, with two setae of unequal length on postero-distal margin, longest seta bifurcate. Merus approximately 3.3 times as long as wide, posterior margin with two long serrate setae (Fig. 5C) centrally, three long serrate setae distally. Carpus with lobe reaching ⅔ length of posterior margin of propodus, anterior margin of lobe with row of serrate setae. Propodus slightly expanding distally, palm convex, nearly transverse, serrate, lined with seven [6 to 9] slender bifurcate spines, corner defined by one stout bifurcate spine, anterior margin with three serrate setae, one near mid-margin and two sub-distal, with one serrate seta distally. Dactylus slightly more than ⅔ length of propodus, slightly exceeding palmar angle, proximal ⅔ of posterior margin serrate. *Medial view* (Fig. 5E): basis with seven bifurcate setae lining anterior and antero-medial margin. Propodus with three serrate setae on inner surface of palm. Dactylus with small seta inserted at distal end of posterior serrate margin.

**Figure 6.**
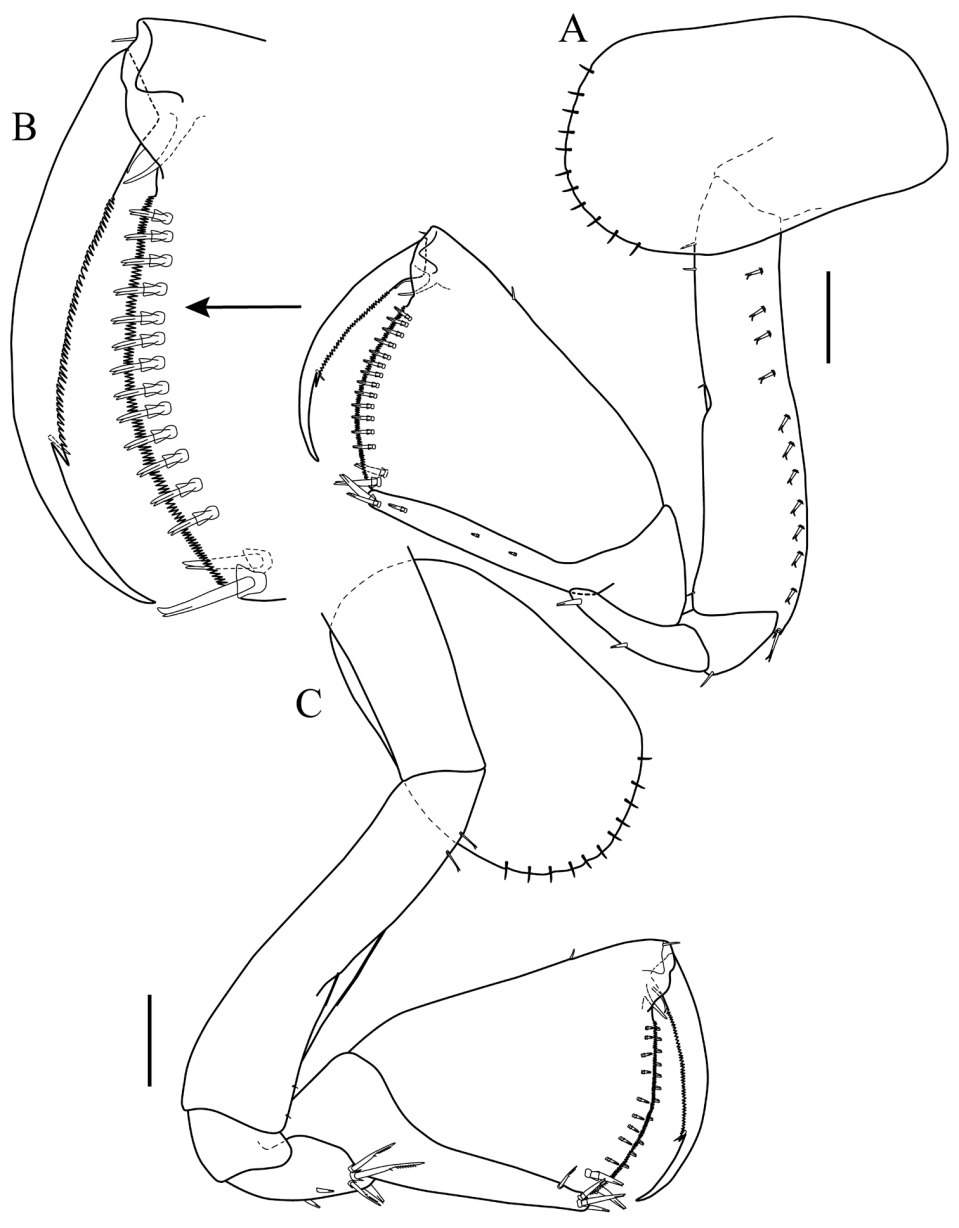
*Apolochus cresti* sp. n., Holotype female: **A** gnathopod 2, lateral view **B** enlargement of tip of propodus and dactylus of gnathopod 2 **C** gnathopod 2, medial view. Scale bars: 0.1 mm (**A, C**).

*Gnathopod 2*: *Lateral view* (Figs 3, 6A−B): sub-chelate; coxal plate sub-rectangular, ventral margin convex, with short marginal setae. Basis approximately 4.8 times as long as wide, anterior margin with four short setae, two proximal, one central, and one distal; posterior margin lined with eleven small setae, one seta distally. Ischium wider than long, with one small seta on postero-distal margin. Merus approximately 4.7 times as long as wide, posterior margin with two setae, one central and one distal. Carpus with elongated lobe reaching along posterior margin of propodus to palmar angle, lateral surface with two small spines and one seta, with two bifurcate setae distally. Propodus slightly expanding distally, palm convex, nearly transverse, serrate, lined with 13 [11 to 14] slender bifurcate spines, corner defined by two stout spines, anterior margin with small spine in distal ⅓, without sub-marginal spines on antero-lateral surface. Dactylus slightly more than ⅔ length of propodus, not quite reaching palmar angle, proximal ⅔ of posterior margin serrate. *Medial view* (Fig. 6C): basis, anterior margin with two spines sub-distally. Merus with three serrate setae on anterior distal margin. Dactylus with small seta inserted at distal end of posterior serrate margin.

**Figure 7.**
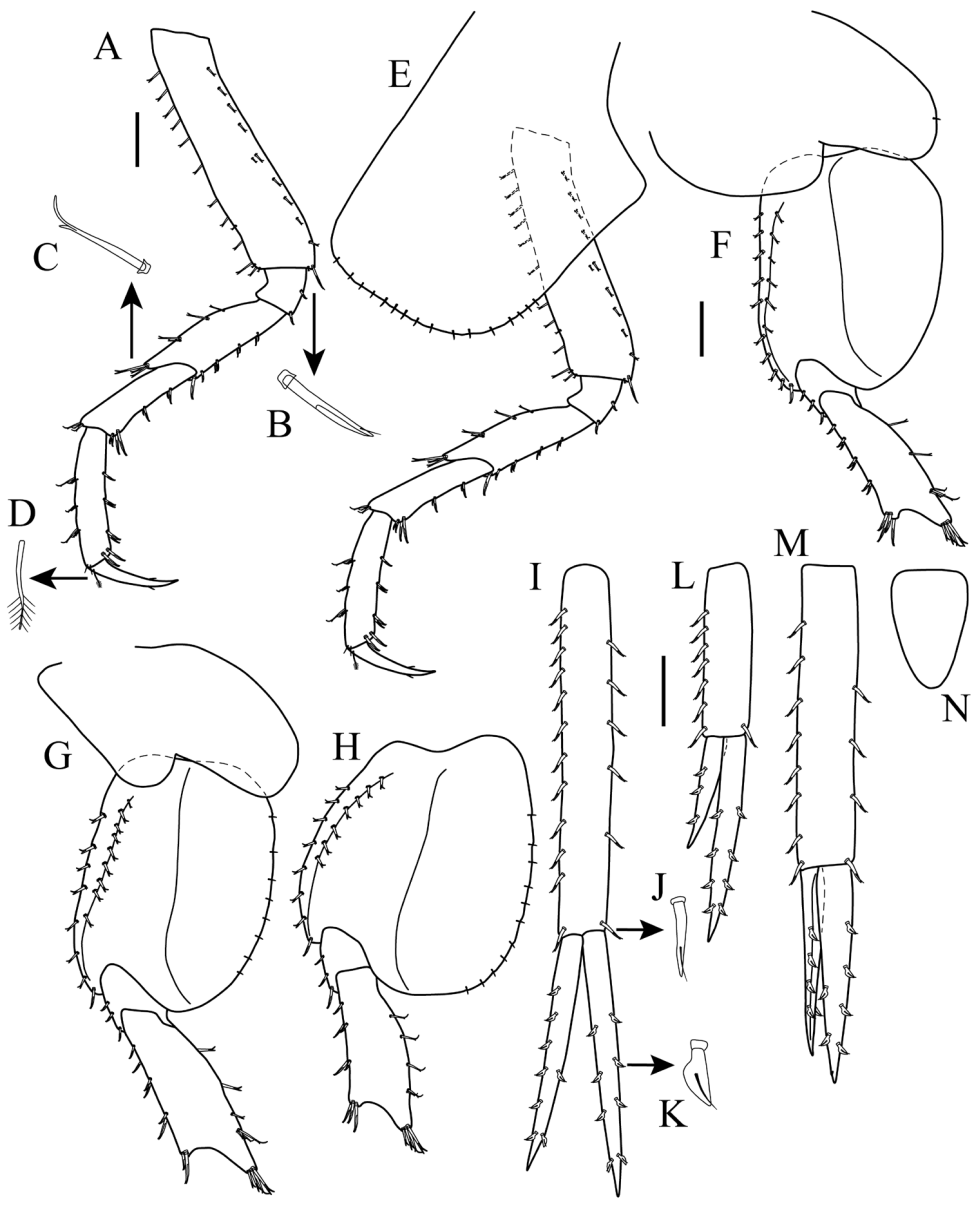
*Apolochus cresti* sp. n., Holotype female: **A** pereopod 3 **B** enlargement of cuspidate seta **C** enlargement of cuspidate seta **D** enlargement of plumose seta **E** pereopod 4 **F** pereopod 5 **G** pereopod 6 **H** pereopod 7 **I** uropod 1 **J** enlargement of cuspidate seta with accessory seta **K** enlargement of robust seta; **L** uropod 2 **M** uropod 3 **N** telson. Scale bars: 1.0 mm (**A, E–H, I, L−N**).

*Pereopod 3* (Figs 2, 7A−D): coxal plate sub-rectangular, longer than wide, ventral margin convex, with short marginal setae (Fig. 2). Basis approximately 4.5 times as long as wide, anterior margin lined with setae of varying lengths, anterodistal margin with four setae of unequal lengths; posterior margin lined with setae of varying lengths, postero-distal margin with one small seta and one cuspidate seta (Fig. 7B). Ischium sub-quadrate, posterior margin with two cuspidate setae, one central and one distal. Merus twice as long as wide, anterior margin with four setae, with three setae of unequal lengths distally; posterior margin with four clusters of two cuspidate setae and one longer cuspidate seta distally. Carpus approximately 3.7 times as long as wide, antero-distal margin with three cuspidate setae (Fig. 7C); posterior margin with two clusters of two cuspidate setae of varying lengths and four cuspidate setae distally. Propodus approximately 5.0 times as long as wide, anterior margin with three clusters of two setae and two simple setae distally; posterior margin with three clusters of two cuspidate setae and two cuspidate setae distally. Dactylus slightly more than 1/2 length of propodus, with plumose seta (Fig. 7D) on antero-proximal margin; with two small setae on each side distally.

*Pereopod 4* (Figs 2, 7E): coxal plate larger than that of coxa 3, quadrate ventral margin straight, with short marginal setae, posterior margin excavate proximally; otherwise as pereopod 3.

*Pereopod 5* (Figs 2, 7F): longer than pereopod 4, coxal plate wider than long, with rounded posterior lobe. Basis expanded, with two longitudinal ridges, anterior margin lined with setae, with cuspidate seta distally, anterior ridge lined with setae. Ischium wider than long, anterior margin lined with three cuspidate setae. Merus approximately 2.9 times as long as wide, posterior margin with two setae, cluster of three setae of unequal length, and four setae distally; anterior margin with one seta, three clusters of two cuspidate setae, with four cuspidate setae distally. Carpus approximately 4.0 times as long as wide, posterior margin with cluster of two setae, with five setae distally; anterior margin with two clusters of two cuspidate setae, with three cuspidate setae distally (Fig. 2). Propodus approximately about 6.2 times as long as wide, posterior margin with four clusters of two setae, with two simple setae distally; anterior margin with three clusters of two cuspidate setae, with two cuspidate setae distally (Fig. 2). Dactylus approximately half length of propodus (Fig. 2A).

*Pereopod 6* (Figs 2, 7G): similar to pereopod 5 except coxal plate smaller than that of pereopod 5. Basis, merus, and propodus slightly longer and more setose.

*Pereopod 7* (Figs 2, 7H): similar to pereopod 6 except coxal plate small and narrowly oval. Basis posterior lobe more expanded. Slightly longer and more setose than pereopod 6.

*Pleon* (Fig. 2): approximately 28% TL, pleonites 1−3 dorsally smooth; *Epimeron 1* (Fig. 2): ventral margin with three spines, postero-ventral corner of plate slightly produced. *Epimeron 2* (Fig. 2): ventral margin with five spines, postero-ventral corner of plate slightly produced. *Epimeron 3* (Fig. 2): ventral margin with four spines, postero-ventral corner of plate sub-quadrate. Pleopods 1−3 (Fig. 2): rami sub-equal in length.

*Urosome* (Fig. 2): approximately 15% TL, urosomites 1−3 dorsally smooth (Fig. 2); *Urosomite 1* longest (Fig. 2); *Urosomite 2* shortest (Fig. 2).

*Uropod 1* (Figs 2, 7I−K): extending beyond peduncle of uropod 3; peduncle longer than rami, inner margin with six cuspidate setae with accessory seta (Fig. 7J), outer margin with 12 [10 to 12] setae cuspidate setae with accessory seta; inner ramus with four inner and four outer robust (Fig. 7K) marginal setae; outer ramus slightly shorter than inner ramus, with two inner and six [4 to 6] outer robust marginal setae; opposing margins of rami setulose (not shown).

*Uropod 2* (Figs 2, 7L): peduncle shorter than peduncles of uropods 1 and 3, inner margin with one distal cuspidate seta with accessory seta, outer margin with seven to eight cuspidate setae with accessory seta; inner ramus with four to five inner and four outer robust marginal setae; outer ramus approximately ⅔ as long as inner ramus, inner margin without setae, outer margin with two to three robust setae; opposing margins of rami setulose (not shown).

*Uropod 3* (Figs 2, 7M): peduncle elongate, shorter than that of uropod 1, inner margin with four cuspidate setae with accessory seta, outer margin with eight cuspidate setae with accessory seta; inner ramus slightly longer than outer ramus, with four inner and two outer robust marginal setae; outer ramus with one inner and four outer robust marginal setae; opposing margins of rami setulose (not shown).

*Telson* (Fig. 7N): sub-triangular, longer than wide, apex rounded.

##### Adult male.

**Figure 8.**
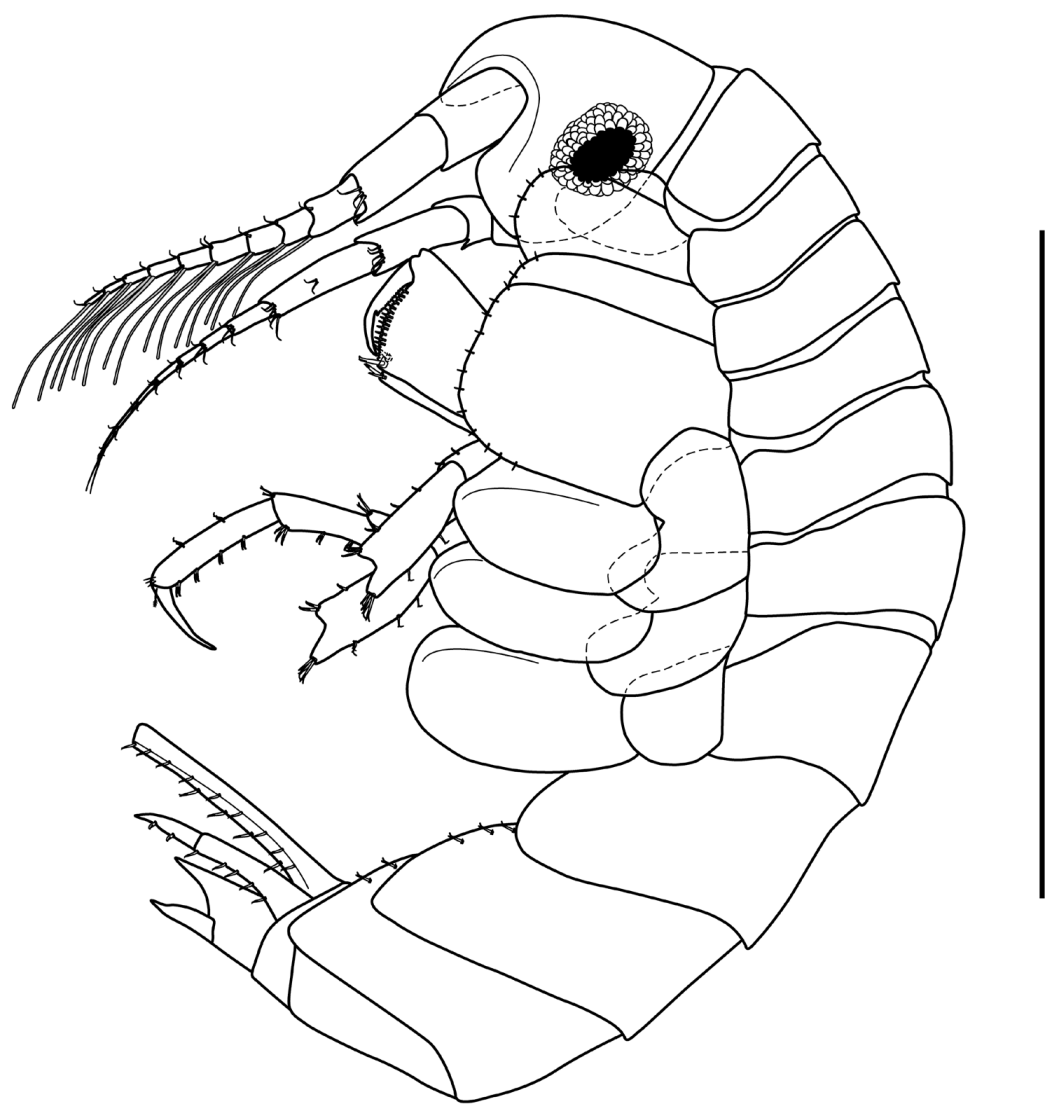
*Apolochus cresti* sp. n., Paratype male: lateral view. Scale bar: 1.0 mm.

*Body* (Fig. 8): Length 2.6 mm, smaller than female; similar to female except for eyes being larger and not completely rounded. Eyes may be darker than those of female (noticed in all males that were found in March 2012). Antenna 1−2 longer than those of female; antenna 1, aesthetascs of flagellum longer and more numerous than those of female.

##### Habitat.

Marine epibenthic, in coastal shallow waters (≤ 2.1 m); *Apolochus cresti* sp. n. was associated with a mixture of macroalgae (e.g., *Agardhiella* sp., *Gracilaria* sp., *Ceramium* sp., and *Cladophora* sp.).

##### Remarks.

Of the eight described species in the genus, *Apolochus cresti* sp. n. can be easily distinguished from *Apolochus barnardi*, *Apolochus casahoya*, the N-E Atlantic *Apolochus neapolitanus* complex (Hoover and Bousfield 2001), *Apolochus delacaya*, *Apolochus picadurus*, and *Apolochus pillai* by having the antenna 1 sub-equal to antenna 2 (antenna 1 is shorter than antenna 2 in the other species). However, *Apolochus cresti*, *Apolochus litoralis*, *Apolochus neapolitanus* from the Mediterranean and *Apolochus staudei* also have antenna 1 sub-equal to antenna 2. *Apolochus cresti* is distinct from *Apolochus litoralis* by (1) the size of the carpal lobe of gnathopod 2 (elongated lobe reaching along posterior margin of propodus in *Apolochus cresti* vs short lobe reaching halfway along posterior margin of propodus in *Apolochus litoralis*), (2) mandible palp article 3 longer than articles 1−2 combined (shorter in *Apolochus litoralis*), and (3) form of the telson (sub-triangular in *Apolochus cresti* vs triangular in *Apolochus litoralis*). The new species differs from *Apolochus staudei* by (1) the size of the accessory flagellum (smaller in *Apolochus cresti*), (2) size and shape of the molar process (bigger and not broadly triangular in *Apolochus cresti*), (3) the number of accessory blades in the spine row of mandible (10−11 vs 15−17, respectively), and (4) the form of the telson (sub-triangular vs narrowly triangular, respectively).

*Apolochus cresti* appears to be most closely related to *Apolochus neapolitanus* (Della Valle, 1893), as figured by Krapp-Schickel (1982) from the Mediterranean, but it can be distinguished by (1) shape of the lateral cephalic lobe (rounded vs truncated, respectively), (2) shape of the molar process (relative strong, with row of spinules/setae running up the margin of the molar column, with three marginal spines on triturating surface in *Apolochus cresti* vs. rounded and rather feeble, lacking marginal setae on column and spines on triturating surface in *Apolochus neapolitanus*), (3) proportion of mandible palp article 3 (longer than previous two articles combined in *Apolochus cresti* vs. shorter than previous two articles combined in *Apolochus neapolitanus*), and (4) gnathopod 2, carpal lobe (just reaching corner of palm in *Apolochus cresti* vs. slightly passing corner of palm in *Apolochus neapolitanus*).

*Apolochus cresti* sp. n. is the fourth species of the genus to be described from the western Atlantic, after *Apolochus casahoya* from Gulf of Mexico, *Apolochus delacaya* from the Gulf of Mexico and Florida Keys, and *Apolochus pillai* from the Florida Keys. *Apolochus cresti* differs from *Apolochus casahoya* and *Apolochus delacaya* by not having antero-lateral spines on the propodus of gnathopod 2 (*Apolochus casahoya* has two spines and *Apolochus delacaya* has four spines). Finally, it is distinct from *Apolochus pillai* in having a long carpal lobe of gnathopod 2, reaching the corner of the palm on the propodus; the carpal lobe is short, not reaching the corner of the palm in latter species. *Apolochus cresti* also differs from *Apolochus* sp. A., which was reported from Florida waters (LeCroy 2002), by having a mandible molar process with a row of spinules/setae running up the margin of the molar column and three marginal spines on the triturating surface versus a mandible molar process with no setae on the margin of the column and a single large apical spine (triturating surface lacking).

The fact that illustrations of *Apolochus neapolitanus* reported around the world are not similar, suggests that it is a complex of cryptic species, or that members of the species have been misidentified due to their small size, fragile body, and difficulty in accessing the mouth parts, which exhibit the most important characters used for taxonomic identification in this group. The following key may be used to further distinguish between the females of known *Apolochus* species.

### Key to the currently recognized species of *Apolochus* (females)

**Table:** 

1	Antenna 1 shorter than antenna 2 (Fig. 9A−D)	**2**
–	Antenna 1 sub-equal to antenna 2 (Fig. 9E−H)	**6**
2	Accessory flagellum lacking (Fig. 9I) or minute (Fig. 9J−K); when it is present, shorter in length than the first article of flagellum (Fig. 9J−K)	**3**
–	Accessory flagellum sub-equal in length to the first article of flagellum (Fig. 9L)	**5**
3	Accessory flagellum lacking (Fig. 9I)	***Apolochus picadurus*** [southern California]
–	Accessory flagellum minute, uniarticulate (Fig. 9J−K)	**4**
4	Gnathopod 2, carpal lobe falling well short of palmar angle (Fig. 9M)	***Apolochus pillai*** [Florida Keys and Gulf of México]
–	Gnathopod 2, carpal lobe nearly reaching palmar angle (Fig. 9N)	***Apolochus barnardi*** [central to southern California]
5	Gnathopod 2, propodus with 1-2 sub-marginal spines on anterolateral surface (Fig. 9O). Uropod 2, peduncle with 1 distal spine on inner margin; outer ramus, lateral margin with 2 stout spines larger than remaining spine (Fig. 9Q)	***Apolochus casahoya*** [Gulf of Mexico, Florida Keys and Venezuela]
–	Gnathopod 2, propodus with 4 sub-marginal spines on anterolateral surface (Fig. 9P). Uropod 2, peduncle without distal spine on inner margin; outer ramus, lateral margin without stout spines, all spines similar in size (Fig. 9R)	***Apolochus delacaya*** [Gulf of Mexico, Florida Keys and Venezuela]
6	Gnathopod 1, carpal lobe reaching halfway along posterior margin of propodus (Fig. 9S−T). Telson narrowly triangular (Fig. 9W–X)	**7**
–	Gnathopod 1, carpal lobe reaching more than halfway along posterior margin of propodus (Fig. 9 U−V). Telson sub-triangular (Fig. 9Y−Z)	**8**
7	Lateral cephalic lobe acute (Fig. 9E)	***Apolochus staudei*** [North Pacific region: British Columbia]
–	Lateral cephalic lobe rounded (Fig. 9F)	***Apolochus litoralis*** [southern California to southern Alaska]
8	Lateral cephalic lobe truncated (Fig. 9G). Mandible molar process rounded, rather feeble, without row of spinules/setae running up the margin of the molar column, without marginal spines on triturating surface (Fig. 9A–1). Mandible palp article 3 sub-equal to articles 1−2 combined (Fig. 9A–1)	***Apolochus neapolitanus* (Della Valle) *sensu* Krapp-Schickel, 1982** [Mediterranean]
–	Lateral cephalic lobe rounded (Fig. 9H). Mandible molar process apex sub-cute, with row of spinules/setae running up the margin of the molar column, with three marginal spines on triturating surface (Fig. 9B–1−B-1’). Mandible palp article 3 longer than articles 1−2 combined (Fig. 9C–1)	***Apolochus cresti* sp. n.** [Mid-Atlantic Region]

**Figure 9.**
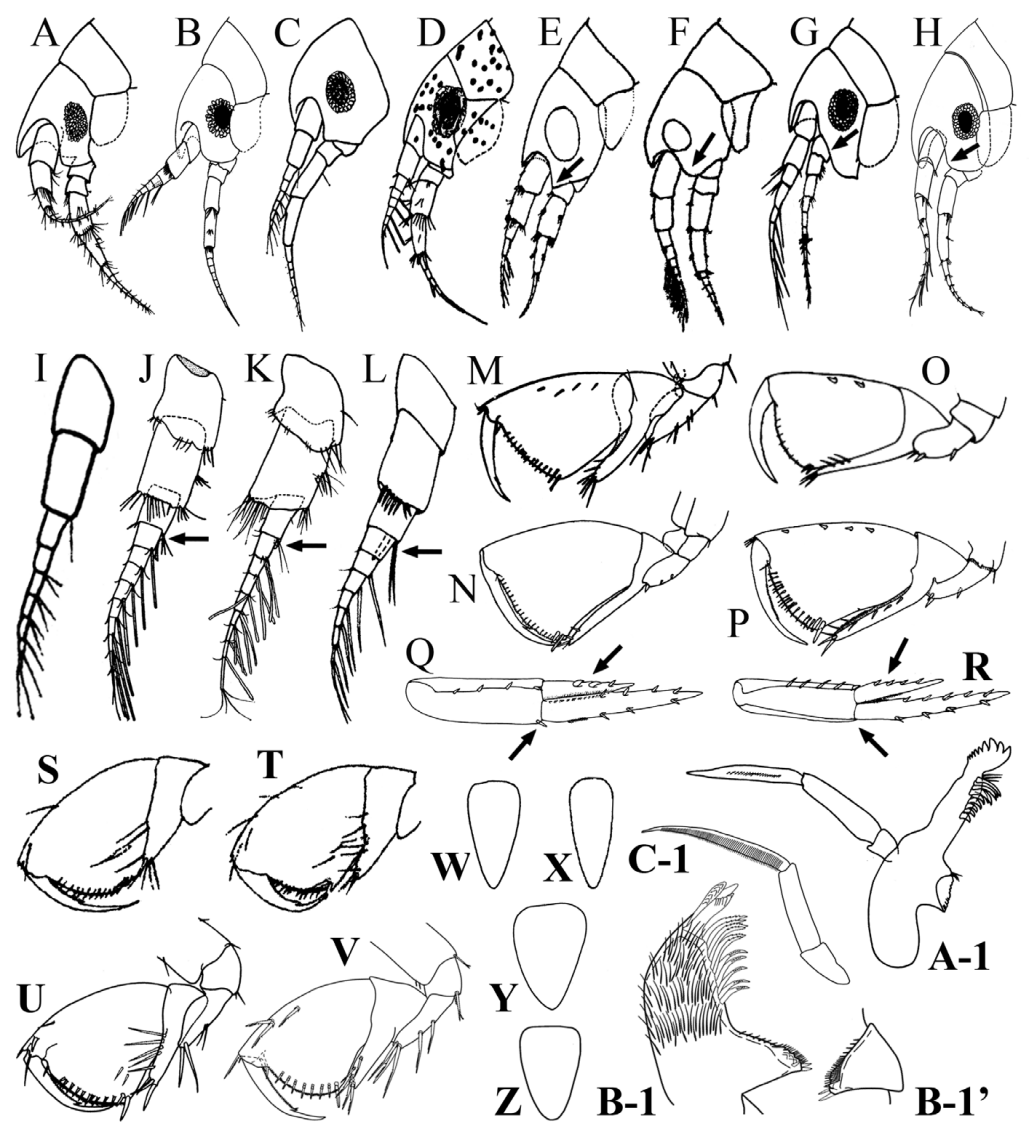
Female head and antennae: **A** *Apolochus barnardi* **B** *Apolochus casahoya* **C** *Apolochus picadurus* **D** *Apolochus pillai* **E** *Apolochus staudei* **F** *Apolochus litoralis* **G** *Apolochus neapolitanus* (*sensu* Krapp-Schickel, 1982) **H** *Apolochus cresti* sp. n. Antenna 1 **I** *Apolochus picadurus* **J** *Apolochus pillai* **K** *Apolochus barnardi* **L** *Apolochus casahoya*. Gnathopod 2: **M** *Apolochus pillai* **N** *Apolochus barnardi* **O** *Apolochus casahoya* **P** *Apolochus delacaya*. Uropod 2: **Q** *Apolochus casahoya* **R** *Apolochus delacaya*. Gnathopod 1: **S** *Apolochus staudei* **T** *Apolochus litoralis* **U** *Apolochus neapolitanus* (*sensu* Krapp-Schickel, 1982) **V** *Apolochus cresti* sp. n. Telson: **W** *Apolochus staudei* **X** *Apolochus litoralis* **Y** *Apolochus neapolitanus* (*sensu* Krapp-Schickel, 1982) **Z** *Apolochus cresti* sp. n. Mandible: **A–1** *Apolochus neapolitanus* (*sensu* Krapp-Schickel, 1982) **B-1**−**B-1** *Apolochus cresti* sp. n. **C-1** *Apolochus cresti* sp. n. [Figures modified from: **A, E, F, K, N, S, T, W**, and **X**, Hoover and Bousfield 2001; **B, L, O−R**, McKinney 1978; **C**, **I**, Barnard 1962; **D**, **J**, and **M**, Barnard and Thomas 1983; **G, U, Y**, and **A–1**, Krapp-Schickel 1982; **H, V, Z** and **B-1−C-1**, Morales-Núñez and Chigbu (this study)]. Not to scale.

### Ecological notes

**Figure 10.**
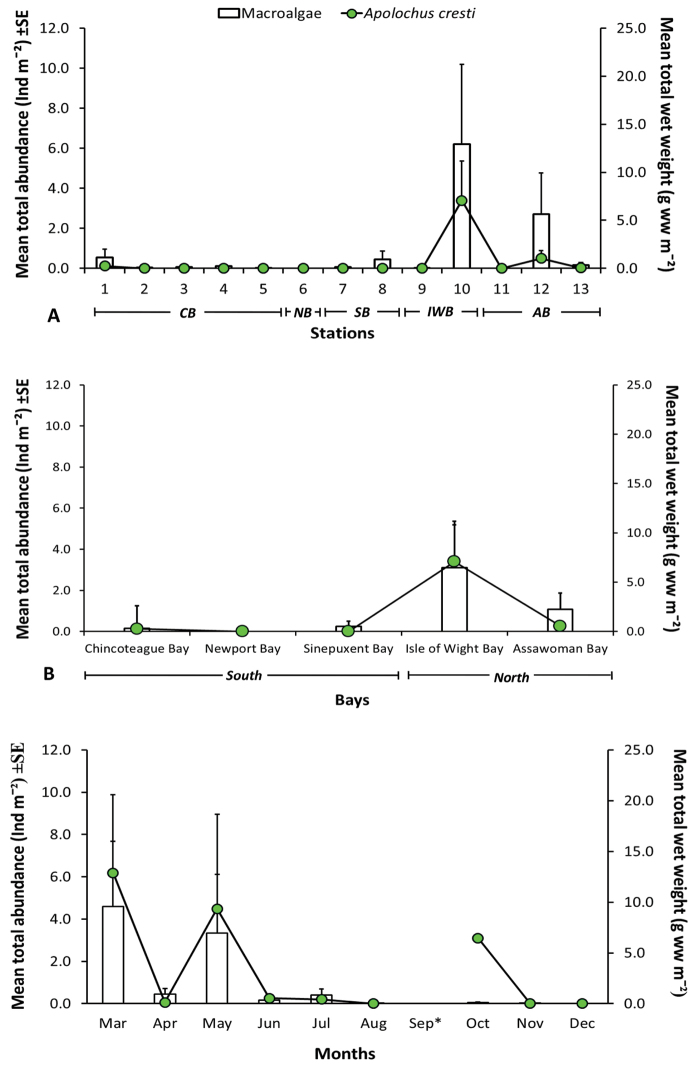
Mean total abundance ± SE of *Apolochus cresti* sp. n. and mean total wet weight of macroalgae ± SE found in Maryland Coastal Bays during this study: **A** stations **B** areas **C** months. CB = Chincoteague Bay; NB = Newport Bay; SB = Sinepuxent Bay; IWB = Isle of Wight Bay; and AB = Assawoman Bay. * Samples were not taken.

A total of 2,105 individuals of *Apolochus cresti* were found in the MCBs. Specimens of *Apolochus cresti* were only found in five of thirteen stations along the bays (Fig. 1). The highest mean abundance of *Apolochus cresti* (3.4 ± 2.0 ind m^-2^) and mean values of macroalgae biomass (12.91 ± 8.33 g ww m^-2^) were found at station 10 in Isle of Wight Bay, in the northern area (Fig. 9A−B, respectively). Spearman’s rank correlation analysis indicated positive correlation between the abundance of *Apolochus cresti* and the amount of macroalgae collected per station (r_s_ = 0.7, p < 0.001), bay (r_s_ = 0.8, p < 0.001), and month (r_s_ = 0.8, p < 0.001) (Fig. 10A−C, respectively). Overall, *Apolochus cresti* was most abundant when a mixture of macroalgae (e.g., *Agardhiella* sp., *Gracilaria* sp., *Ceramium* sp., and *Cladophora* sp.) was observed in Isle of Wight and Assawoman Bays in the northern area of MCBs during March in this study. Furthermore, no significant correlations (p > 0.05) were observed between the abundance of *Apolochus cresti* and abiotic variables measured in the bays (Tables 1–2).

**Figure 11.**
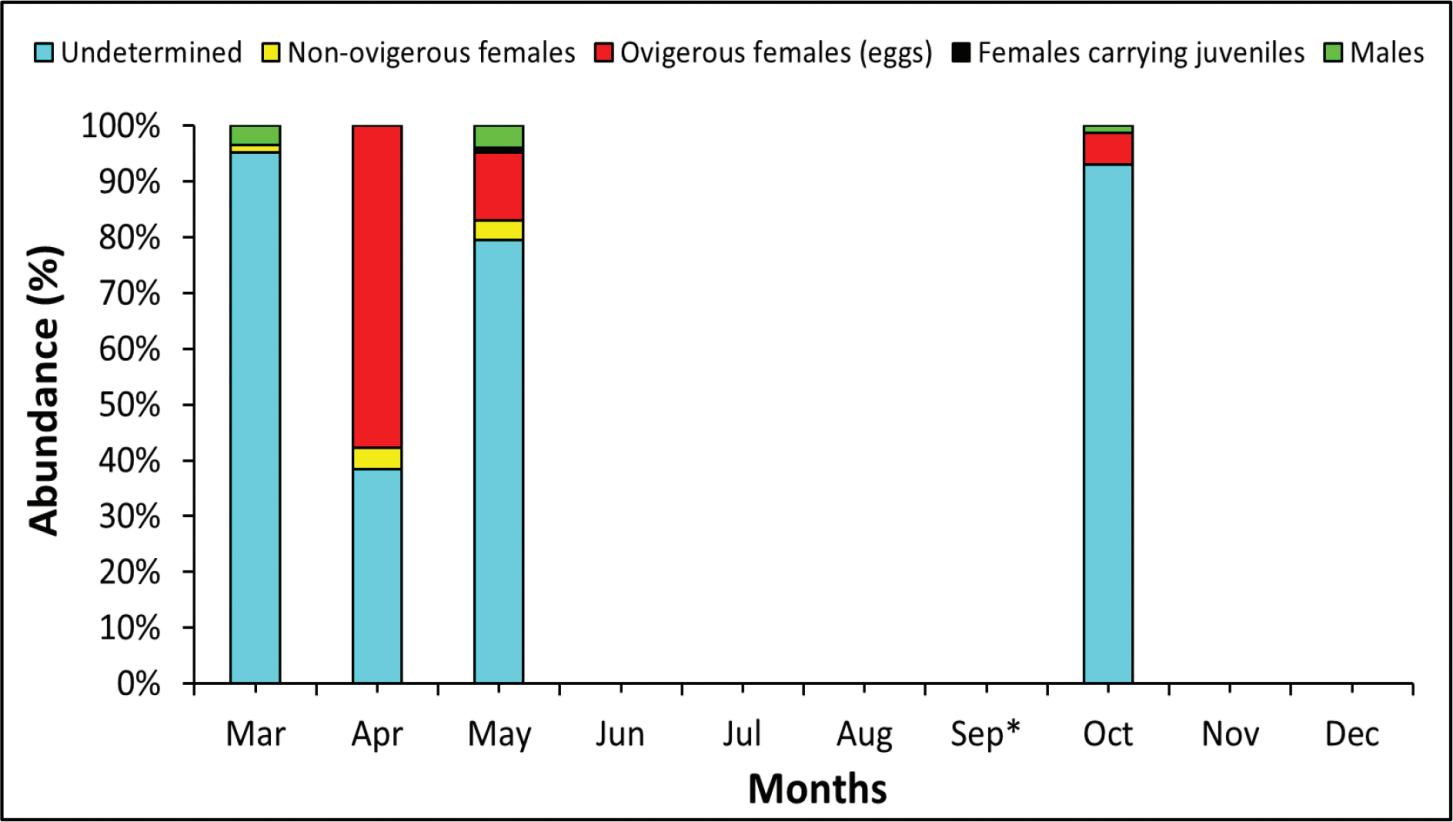
Percentage of undetermined, non-ovigerous females, ovigerous females and males of *Apolochus cresti* sp. n. found in Maryland Coastal Bays during this study. * Samples were not taken.

The catch per effort of non-ovigerous females decreased from 11 (March) to 1 (April) before increasing to 22 (may). However, the relative abundance of non-ovigerous females in the population was similar (~3.5 %) in April and May (Fig. 11). Ovigerous females carrying eggs were present from March (just one specimen) to May, and in October, reaching their peak in May (78). Conversely, the higher relative abundance of females carrying eggs was found in April (Fig. 11). Females carrying juveniles were only found in May (Fig. 11). Males were abundant in March and May, and were also collected in October (Fig. 11).

## Supplementary Material

XML Treatment for Apolochus

XML Treatment for Apolochus cresti
